# Bariatric Surgery and Brain Health—A Longitudinal Observational Study Investigating the Effect of Surgery on Cognitive Function and Gray Matter Volume

**DOI:** 10.3390/nu12010127

**Published:** 2020-01-02

**Authors:** Kristin Prehn, Thorge Profitlich, Ida Rangus, Sebastian Heßler, A. Veronica Witte, Ulrike Grittner, Jürgen Ordemann, Agnes Flöel

**Affiliations:** 1 Department of Neurology & NeuroCure Clinical Research Center, Charité—Universitätsmedizin Berlin, 10117 Berlin, Germany; 2 Department of Psychology, Medical School Hamburg, 20457 Hamburg, Germany; 3 Department of Neurology, Aging and Obesity Group, Max Planck Institute for Human Cognitive and Brain Sciences, 04103 Leipzig, Germany; 4 Institute of Biometry and Clinical Epidemiology, Charité—Universitätsmedizin Berlin, 10117 Berlin, Germany; 5 Berlin Institute of Health, Charité—Universitätsmedizin Berlin, 10117 Berlin, Germany; 6 Center for Bariatric and Metabolic Surgery, Charité—Universitätsmedizin Berlin, 10117 Berlin, Germany; 7 Center for Bariatric and Metabolic Surgery, Vivantes Klinikum Spandau, 13585 Berlin, Germany; 8 Department of Neurology, University of Greifswald, 17489 Greifswald, Germany; 9 German Center for Neurodegenerative Diseases, Standort Rostock/Greifswald, 17489 Greifswald, Germany

**Keywords:** Roux-en-Y gastric bypass, executive functions, voxel-based morphometry, fronto-temporal cortex, nucleus accumbens

## Abstract

Dietary modifications leading to weight loss have been suggested as a means to improve brain health. In morbid obesity, bariatric surgery (BARS)—including different procedures, such as vertical sleeve gastrectomy (VSG), gastric banding (GB), or Roux-en-Y gastric bypass (RYGB) surgery—is performed to induce rapid weight loss. Combining reduced food intake and malabsorption of nutrients, RYGB might be most effective, but requires life-long follow-up treatment. Here, we tested 40 patients before and six months after surgery (BARS group) using a neuropsychological test battery and compared them with a waiting list control group. Subsamples of both groups underwent structural MRI and were examined for differences between surgical procedures. No substantial differences between BARS and control group emerged with regard to cognition. However, larger gray matter volume in fronto-temporal brain areas accompanied by smaller volume in the ventral striatum was seen in the BARS group compared to controls. RYGB patients compared to patients with restrictive treatment alone (VSG/GB) had higher weight loss, but did not benefit more in cognitive outcomes. In sum, the data of our study suggest that BARS might lead to brain structure reorganization at long-term follow-up, while the type of surgical procedure does not differentially modulate cognitive performance.

## 1. Introduction

Obesity and overweight have been increasing dramatically in most countries around the world and constitute a major health problem [1]. In particular, obesity increases the risk for diseases, such as type 2 diabetes, hypertension, coronary heart disease, and stroke [2]. Moreover, it is associated with the reduced cognitive performance [3,4], and even with dementia in later life [5].

Conversely, dietary modifications leading to weight loss have been suggested as a means to improve brain health (for reviews and putative cellular mechanisms, such as the engagement of adaptive cellular stress response pathways, see Reference [6,7]). Evidence from studies in humans, however, is scarce (for a review and metaanalysis, see Reference [8]). In a previous study [9], we found that intense weight loss after a low caloric diet in older women with obesity was associated with improved cognition (e.g., executive functions) paralleled by increased gray matter (GM) volume in inferior frontal gyrus and hippocampus.

One highly effective means leading to rapid and sustainable weight loss in obesity is bariatric surgery (BARS). BARS leads to an average long-term weight loss of 10–25% [10], and thus, surpasses other treatment opportunities, such as nutritional counseling and sport interventions [11]. Approaches include gastric banding (GB), vertical sleeve gastrectomy (VSG), and gastric bypass surgeries, most often the Roux-en-Y gastric bypass (RYGB). While GB and VSG only restrict the amount of food that can be consumed (purely restrictive treatment), an RYGB is built to circumvent large parts of the gastrointestinal tract [12], and leads to both restrictions of food intake and malabsorption of nutrients (combined restrictive-malabsorptive treatment). In addition to weight loss and recovery from obesity-related diseases, a number of studies showed improved cognitive function after BARS (e.g., References [13,14,15,16]). Although most studies documented improvement in at least one neurocognitive domain, such as executive functions, memory, or attention, they were not unequivocal and consistent (for reviews, see References [17,18,19,20]). Georgiadou et al. [21], for instance, was unable to find any substantial differences in cognitive assessments comparing 50 post-RYGB patients to a well-matched sample of patients seeking BARS. Although patients treated with a combined restrictive-malabsorptive approach (i.e., RYGB) show greater and faster weight loss, as well as faster improvement in glucose control than patients treated with purely restrictive procedures (i.e., VSG and GB; [22]), it is unclear whether this approach also benefits patients more with regard to brain health. For instance, it has been suggested that the surgical removal of parts of the gut with hormonal activity might also affect brain structure and function [23], (for a review, see Reference [24]). Smith et al. [25] compared cognitive functions in RYGB and VSG patients before and after surgery and found improvements in executive functions and processing speed in both groups. RYGB patients also exhibited improved attention, but no group × time interactions were reported.

In the present study, we tested cognitive performance in patients before and six months after BARS using a comprehensive neuropsychological testing battery (primary outcome parameter: Composite executive functions score; see Reference [9]). The same cognitive performance test was done in a patient group waiting for BARS at similar time points. We expected that patients undergoing BARS compared to controls would show improved executive control together with further improvements in memory, mood/affect and health-related quality of life [9]. Since intervention-induced regional changes in GM volume have been found to correlate and precede possible improvement in cognitive functions [9,26,27,28], GM volume was measured at both time points in all patients eligible for magnetic resonance imaging (MRI) as a task-independent sensitive and reliable biomarker. In particular, we expected that BARS patients would show an increase of GM volume in the fronto-temporal cortex [9,29]. Finally, we explored the question of differences in cognitive outcomes between surgical procedures.

## 2. Materials and Methods

### 2.1. Study Overview

**Figure 1:**
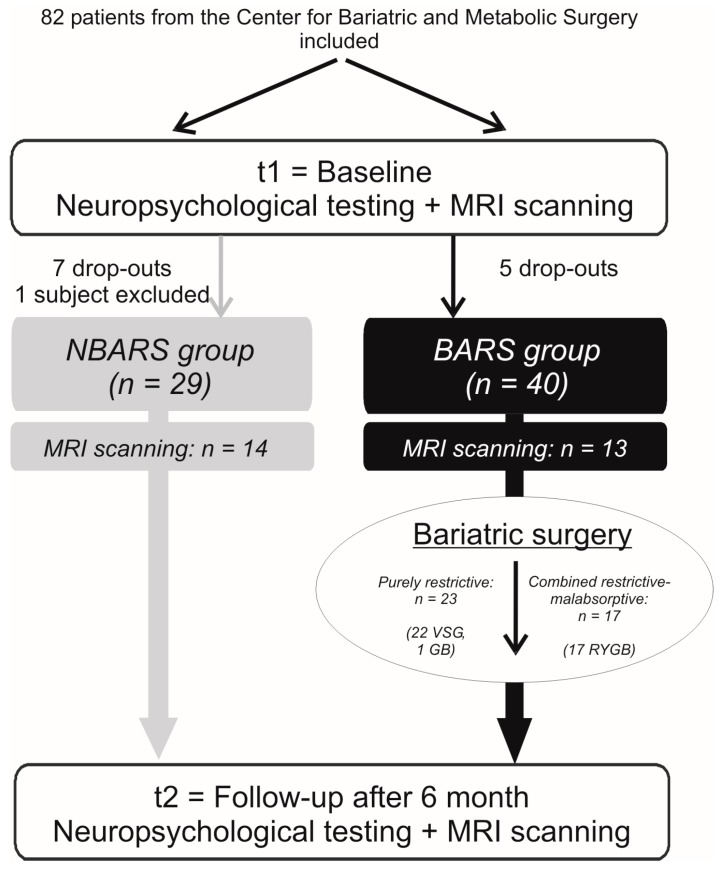
Study flow. Eighty-two patients with obesity were recruited and assigned to bariatric surgery (BARS) or waiting for control group (NBARS group) based on whether they had already been scheduled for surgery, or whether they could not be scheduled within the next six months, respectively. Forty patients in the BARS (28 women, mean age = 46 ± 11 years) and 29 patients in the NBARS group (16 women; mean age = 45 ± 12 years) underwent baseline and follow-up measurements, including medical examination and neuropsychological testing. Thirteen patients in the BARS and 14 patients in the NBARS group also underwent structural MRI. We also looked for effects of surgical procedures and divided surgery patients into two groups [purely restrictive treatment (*n* = 23): VSG/GB group; combined restrictive-malabsorptive treatment (*n* = 17): RYGB group].

The study employed a prospective and longitudinal observational study design and was conducted at Charité—Universitätsmedizin Berlin. Forty patients scheduled for BARS (BARS group) were tested before (t1) and six months after the intervention (t2) in a range of cognitive domains, including executive functions (primary outcome), memory, and attention and compared to a waiting list control group at similar time points (NBARS group; *n* = 29). From these 69 patients, 27 were also eligible for structural magnetic resonance imaging (MRI; BARS group: *n* = 13, NBARS group: *n* = 14) at both time points. In addition, blood samples were taken, and anthropometric data, such as height, weight, blood pressure, and body fat percentage were recorded. In a second step, BARS patients were compared with regard to whether surgical procedures were purely restrictive (VSG/GB group, *n* = 23) or combined restrictive-malabsorptive (RYGB group, *n* = 17). For an overview of the study flow, see Figure 1.

### 2.2. Participants

All participants were recruited from the Center for Bariatric and Metabolic Surgery at Charité—Universitätsmedizin Berlin. Inclusion criteria comprised age between 18 and 70 years and formal requirements for BARS according to the BARS guidelines: Patients must either have (1) a BMI greater than 40 kg/m^2^ and failure of conservative obesity treatment or (2) a BMI greater than 35 kg/m^2^, at least one typical co-morbidity (such as type-2 diabetes, hypertension, non-alcoholic fatty liver disease), and failure of conservative obesity treatment. Exclusion criteria included the history of severe untreated medical, neurological, and psychiatric diseases, brain pathologies identified in the MRI scan, and non-fluent German language abilities. A mini mental state examination (MMSE; [30]) score below 24, indicating cognitive impairment [31], was also set as an exclusion criterion.

Since a sample size calculation conducted prior to the study revealed that 40 subjects per group are needed to detect a difference in the primary outcome parameter between the groups at t2 with moderate effect size, 82 subjects were recruited into the study (based on inclusion and exclusion criteria detailed above). Subjects recruited into the study were either assigned to the BARS or to the NBARS group based on whether they had already been scheduled for surgery, or whether they could not be scheduled within the next six months, respectively. The waiting time of more than six months in the NBARS group gave us the time to carry out baseline and follow-up measurements before surgery. Although patients were not randomly assigned, groups did not differ with regard to key baseline characteristics. First, all patients included into the study were eligible for bariatric surgery: After careful evaluation by the treating physicians (psychiatrist and surgeon), waiting list group patients had been recommended to undergo bariatric surgery, but had to wait for this procedure, due to the prolonged process of receiving approval by health insurances in Germany. Second, demographic and health-related parameters, such as age, gender, educational level, verbal intelligence, and depression score were comparable, except for BMI which was 4 kg/m^2^ higher in the BARS group (see Section 3 on the analysis of baseline characteristics and Table 1(a)).

Unfortunately, the recruitment of patients to the NBARS group from the Center for Bariatric and Metabolic Surgery turned out to be more difficult than expected, since most patients interested in participating were already scheduled for surgery. Additionally, we were confronted with a rather high number of participants that dropped out of the study, especially in the NBARS group. Five patients in the BARS and seven patients in the NBARS group did not complete the study, due to personal reasons, such as time constraints or change of residence. Finally, we had to exclude one additional patient from the waiting list control group, because he changed his lifestyle quite markedly, thus, lost more than 15% of his initial weight during the waiting period and was no longer eligible for BARS, leaving only 69 participants for the analysis (BARS group: *n* = 40, 28 women; NBARS group: *n* = 29, 16 women).

MRI scanning was possible in 13 patients of the BARS and 14 patients of the NBARS group. The remaining patients did not tolerate scanning, either due to claustrophobia or to the size of the MR bore (diameter of 60 cm only).

From the 40 patients in the BARS group, 17 were treated with RYGB (combined restrictive-malabsorptive or RYGB group), while 23 patients underwent VSG (*n* = 22) or GB (*n* = 1), and thus, were assigned to the “purely restrictive” or VSG/GB group.

The research protocol was approved by the local Institutional Review Board of the Charité—Universitätsmedizin Berlin (ethical approval code: EA1/074/11). The study was carried out in accordance with the principles of the Declaration of Helsinki. All subjects provided written informed consent before the investigation and received a small reimbursement for their participation.

### 2.3. Assessment of Neuropsychological and Physical Data

Assessment of neuropsychological and physical data at t1 and t2 was done by a research assistant trained in clinical neuropsychology. Subjects arrived in the morning (between 07:00 and 12:00) after an overnight fast. First, they underwent a standardized medical interview, including a neurological examination. After blood sampling and measurement of anthropometric parameters (i.e., body weight, height, BMI, systolic and diastolic blood pressure, body fat percentage), subjects were given around one hour time for breakfast. After the break, they were tested using a comprehensive test battery with a focus on executive functions (primary outcome parameter), memory, sensomotor speed, and attention. Finally, MRI scanning was done, if possible, despite high weight (patients were not eligible for the MRI part of the study if contraindications for MRI were present).

For measuring executive functions, the test battery included phonematic and semantic verbal fluency [32], the trail making test (TMT) part A and B [33], and the Stroop color-word interference test [17]. Memory was assessed with the German version of the auditory verbal learning test (VLMT; [18]) and digit span backwards. Subscores of the TMT, Stroop, and the digit span task were used to provide measures of sensomotor speed and attention.

In particular, phonematic verbal fluency was measured by the average number of words with the initial letter “S” and “P” a subject was able to produce in one minute. To measure semantic fluency, the categories “animal species” and “given names” were chosen.

The trail making test (TMT) comprised two parts. In part A, subjects were instructed to connect numbers from 1 to 25 on a sheet of paper in sequential order as quickly as possible. In part B, subjects had to alternate between numbers and letters (1, A, 2, B, etc.). The time to complete each part was taken as testing scores.

The Stroop (color-word interference) test comprised three parts. In part 1, subjects had to read as quickly as possible a list of color names (“red,” “yellow,” “green,” and “blue”). In part 2, subjects had to identify the color of printed bars. In part 3, subjects were presented again with a list of color names. These names, however, were not printed in black, but colored (red, yellow, green, and blue) ink in a way that the ink color never corresponded to the meaning of the word (e.g., “red” printed in green) and subjects had to identify the ink color. time to complete each part was taken as testing scores.

In the VLMT, subjects had to learn as many words as possible from a spoken list of 15 words presented in five consecutive trials. Subjects had to recall the words immediately after each trial and after a 30 min delay. Finally, subjects had to identify the previously learned words from a list which also included 20 new words and 15 words from an interference list learned during the delay. In sum, the VLMT allows for the calculation of at least three different memory scores: The “VLMT learning score” is defined as the sum of correctly recalled words during the five immediate learning trials (maximum—75 words). The number of correctly recalled words after the 30 min delay (maximum—15 words) is taken as the “VLMT delayed recall score.” Finally, the “VLMT recognition score” comprises the number of correctly recognized words minus false positive identifications (maximum—15 words). To avoid test-retest effects, two parallel versions of the VLMT were used and counterbalanced across groups and time points.

Digit span for- and backwards was measured by the number of digits a subject was able to recall in correct or reversed order immediately.

Test scores were z-transformed and averaged to create composite scores for each of the cognitive domains according to van de Rest et al. [34]:

1. Executive functions = [z_phonematic fluency_ + z_semantic fluency_ − z_TMT (part B − part A)/part A_ − z_Stroop (part 3 − (part 1 + part 2))/2_]/4;
2. Memory = (z_VLMT learning score_ + z_VLMT delayed recall score_ + z_VLMT recognition score_ + z_digit span backwards_)/4;
3. Sensomotor speed = (−z_TMT part A_ − z_Stroop part A_ − z_Stroop part B_)/3;
4. Attention = z_digit span forwards_.

Changes in affect and mood-related to BARS were also assessed using the Positive and Negative Affect Schedule (PANAS; [35]) and the State-Trait Anxiety Inventory (STAI; [36]) at t1 and t2. Changes in health-related quality of life were assessed with a 12-item short-form health survey resulting in a physical and psychological subscore (SF-12; [37]). To monitor how participants changed physical activity over time, which might by itself influence brain structure and function [38], we used the Freiburger Questionnaire on Physical Activity (FKA; [39]) at both time points. In addition, psychiatric co-morbidity was monitored using the Beck’s Depression Inventory (BDI; [40]). Only at t1, subjects were also screened for cognitive impairment using the MMSE and general verbal intelligence was measured using the multiple choice vocabulary test (MWT-B; [41]).

Body fat percentage was computed via bioelectric impedance data analysis performed with B.I.A. 2000-M (Data Input GmbH, Pöcking, Germany) and the software NutriPlus (Data Input GmbH, Pöcking, Germany).

Serum levels of glycated hemoglobin A1c (HbA1c), fasting glucose and insulin, triacylglycerides, total cholesterol, high-to-low density lipoprotein (HDL-to-LDL) ratio, tumor necrosis factor-alpha (TNF-α), interleukin-6, high-sensitive C-reactive protein (hsCRP), and leptin were analyzed by the IMD laboratory, Berlin, Germany. The HOMA index was calculated from fasting glucose and insulin levels to indicate insulin resistance.

### 2.4. Statistical Analysis of Neuropsychological and Physical Data

Statistical analysis of neuropsychological data and physical parameters (was performed using SPSS 23.0 (PASW, SPSS; IBM, Armonk, NY, USA). In the case of skewed distributions (|skewness| > 1), variables were rank-transformed.

To assess changes in cognitive abilities, mood/affect, health-related quality of life, blood levels, and anthropometric data over time in subjects of the BARS compared with the waiting list control group, we computed difference scores for each subject between t1 and t2. Difference scores between groups were then compared using ANCOVAs with the group allocation (BARS vs. NBARS group and RYGB vs. VSG/GB group, respectively) as the independent variable. To control for interindividual variability, baseline score values were entered as covariates. To account for differences in age and BMI before the intervention, age and BMI at baseline were also added as covariates in all analyses. We additionally tested for changes over time within each group and calculated post-hoc paired t-tests for illustration purposes (t1 vs. t2).

Since baseline function measures usually have a high impact on follow-up measures, we tested group x baseline value interaction terms on the executive functions change score (primary outcome) and found a significant baseline value × group interaction, indicating that patients with lower baseline scores showed a greater improvement after the intervention than patients with good performance at baseline (see in detail Section 3.2). As reported in the Section 3, we found no group x time interaction with regard to the primary outcome in the main analysis, supporting the hypothesis that BARS compared to NBARS patients improved in executive control. The significant baseline value x group interaction, however, suggested that our hypothesis might still be true for poor performers. Therefore, exploratory post-hoc analyses were computed analyzing marginal effects in: (1) Patients who scored high; (2) average; and (3) low in executive functions tests at t1 using tertiles of the score.

Finally, to explore the question of whether improvements in executive functions were related to changes in insulin-glucose metabolism or inflammatory markers, bivariate correlation analyses were run.

The level of significance was set at α = 0.05 (two-tailed) for analyzing the primary outcome (composite executive functions score). All secondary analyses were done exploratory. For all ANCOVAs, we report regression coefficients ß, 95% confidence intervals (CI), η_p_^2^ (partial eta squared) as measures of effect sizes, and *p*-values. For analyses of marginal effects, we report mean differences (I–J), 95% CIs, η_p_^2^ as measures of effect sizes, and *p*-values. No adjustments to control for the number of comparisons, due to multiple hypotheses testing were conducted for the secondary analyses.

### 2.5. MRI Data Acquisition and Voxel-Based Morphometry

MRI was performed on a Siemens Trio system operating at 3 T and using a 12-channel head coil. The anatomical scan consisted of 192 slices and was acquired in the sagittal plane using a high-resolution T1-weighted magnetization-prepared rapid acquisition with gradient echo (MPRAGE) sequence (TR = 1900 ms, TE = 2.52 ms, a = 9°, voxel size = 1 × 1 × 1 mm^3^).

Analysis of high-resolution anatomical images was conducted using the SPM8 voxel-based morphometry (VBM) toolbox (VBM8; http://dbm.neuro.uni-jena.de/vbm). Since the analysis of longitudinal anatomical data requires customized processing that considers differences within each individual separately, data pre-processing was done using a specific batch for longitudinal data analysis (method as described in Reference [9]). This batch registers baseline and follow-up images of each subject to the mean of both images and calculates structural differences (i.e., intra-individual changes) by applying spatial normalization parameters, which were estimated during segmentation of the mean image, to both images. In detail, data pre-processing with VBM8 comprised the following steps for each subject: First, baseline and follow-up images (t1, t2) were initially realigned to a T1 template in MNI space. Second, a mean image (averaged from t1 and t2) was calculated and raw data (t1 and t2) were realigned using the mean image as the reference image. Third, images were bias-corrected to account for signal inhomogeneities, and in the next step, segmented into the different tissue classes (GM, white matter, cerebrospinal fluid). This segmentation procedure was further refined (1) by accounting for partial volume effects [42], (2) by using adaptive maximum a posteriori estimations [43], and (3) by applying a hidden Markov random field model [44]. The resulting tissue maps were spatially normalized using a specific MNI template derived from 550 healthy control subjects of the IXI database (http://www.brain-development.org) and linear (12-parameter affine) transformations together with a non-linear diffeomorphic image registration algorithm (DARTEL; [45]). Spatial normalization parameters obtained from the segmented mean image were finally applied to the segmentation of the bias-corrected baseline and follow-up images, which were realigned again. Data were not modulated (i.e., scaled by the amount of contraction or expansion during normalization), because scaling is not necessary for longitudinal studies in which the focus is on relative differences between two images of the same participant [46]. In the last step, GM segments (wp1mr*) representing GM volume or density, respectively, were smoothed with a 10 mm full-width-at-half-maximum (FWHM; [47]) Gaussian kernel suitable for small sample sizes [9,48].

For the statistical analysis of VBM data (i.e., individual wp1mr* GM segments), we used repeated-measures ANOVAs with a flexible factorial design comprising the factors “subject,” “group,” and “time” testing for group x time interactions as implemented in SPM8. Since it has been found that image artifacts inducing head motion significantly decrease with changes in BMI [49,50,51], head motion measured as mean framewise displacement at both time points (t1 and t2) was entered as a covariate of no interest. To infer changes in GM volume in the BARS compared to the NBARS group between t1 and t2, in each analysis two t-contrasts were formulated: (1) BARS group > NBARS group^t2>t1^ (indicating an increase in GM volume in the BARS group in comparison with the NBARS group) and (2) NBARS group > BARS group^t2>t1^ (indicating a decrease in the BARS group in comparison with the NBARS group). After significant group × time interactions were detected, we also looked at changes in GM volume within each group separately (contrasts: BARS group^t2>t1^, BARS group^t1>t2^, NBARS group^t2>t1^, NBARS group^t1>t2^).

In the analysis of GM volume changes, absolute GM thresholds of 0.2 were used to prevent edge effects located at the border regions of the tissue maps. Reported changes had *p*-values < 0.05 after cluster-wise FWE correction at a cluster-defining threshold of *p* < 0.005, uncorrected.

For illustrative purposes, we extracted GM values in arbitrary units within the two clusters identified in the whole brain analysis (a cluster in the left superior temporal gyrus extending to insula and inferior frontal gyrus and a cluster in the bilateral ventral striatum) using the MarsBaR toolbox for SPM (marsbar.sourceforge.net).

## 3. Results

### 3.1. Baseline Characteristics

Subjects in the BARS and NBARS groups were comparable with regard to age, gender, years of education, general verbal intelligence measured with the vocabulary test, depression score assessed with the BDI, and levels of fasting glucose and HbA1c (indicating the level of diabetes; see Table 1(a), for demographic and baseline characteristics). Subjects in the BARS group, however, had on average a 4.6 kg/m^2^ higher BMI than subjects in the NBARS group [95% CI = (1.17, 7.94), t(67) = 2.69, *p* = 0.01].

In addition to the main BARS vs. NBARS comparison, we compared two subsamples of patients (1) patients in the BARS and NBARS group who also underwent structural MRI and (2) patients in the BARS group that underwent a purely restrictive (i.e., VSG/GB group) or combined restrictive-malabsorptive surgical procedure (i.e., RYGB group). Subjects in both MRI samples were comparable in demographics and baseline characteristics (see Table 1(b)). Subjects in both surgery groups differed in the percentage of women [57% in VSG/GB vs. 88% in RYGB, χ2(1) = 4.68, *p* = 0.03], but were comparable with regard to all other demographic and baseline characteristics (see Table 1(c)).

### 3.2. Changes in Physical and Neuropsychological Parameters

Several physical parameters were improved in the BARS group as compared to controls six months after surgery. That is, we found group × time interactions in the ANCOVAs, indicating lower weight and BMI, body fat proportions, as well as a systolic and diastolic blood pressure (for descriptive statistics, regression coefficients ß, 95% Cis, ηp2s, and *p*-values, see Table 2).

Additional within group comparisons indicated changes of physical measures in the BARS group, while same parameters in the NBARS group did not change substantially.

With regard to serum parameters, we found group × time interactions indicating changes over time in the BARS compared to the NBARS group. In particular, glucose metabolism improved (lower fasting glucose, fasting insulin, HOMA-index) and triacylglycerides, markers of inflammation (IL-6 and hsCRP), and leptin levels decreased. Within group comparisons showed that all serum parameters changed in the BARS group, whereas, no substantial change or even an unexpected increase (in leptin levels) occurred in the NBARS group (see Table 2).

With regard to the primary outcome (composite executive functions score), we found no significant group × time interaction (see Table 2). However, exploratory within group comparisons showed improvement of executive functions in the BARS group over time [mean difference = 0.26, 95% CI = (0.07, 0.45), t(33) = −2.75, *p* = 0.01], which was not evident in the NBARS group [mean difference = −0.05, 95% CI = (−0.22, 0.11), t(26) = 0.67, *p* = 0.51]. We also found no substantial group × time interactions for secondary cognitive outcomes. However, ANCOVAs showed an impact of baseline values at t1 on cognitive changes (i.e., difference scores) in the domains memory [ß = −0.72, 95% CI = (−1.1, −0.4), η_p_^2^ = 0.21, *p* < 0.001], sensomotor speed [ß = −0.3, 95% CI = (−0.6, −0.0), η_p_^2^ = 0.07, *p* = 0.04], and attention [ß = −0.6, 95% CI = (−0.9, −0.3), η_p_^2^ = 0.20, *p* < 0.001], and in addition, a baseline value × group interaction in the cognitive domain executive functions [ß = −0.39, 95% CI = (−0.7, −0.1), η_p_^2^ = 0.10, *p* < 0.02]. This negative relationship (indicated by a negative ß value) shows that patients with higher executive functions test scores at t1 showed worse performance at t2, while patients with lower scores improved after surgery in contrast to subjects in the waiting control group.

**Figure 2:**
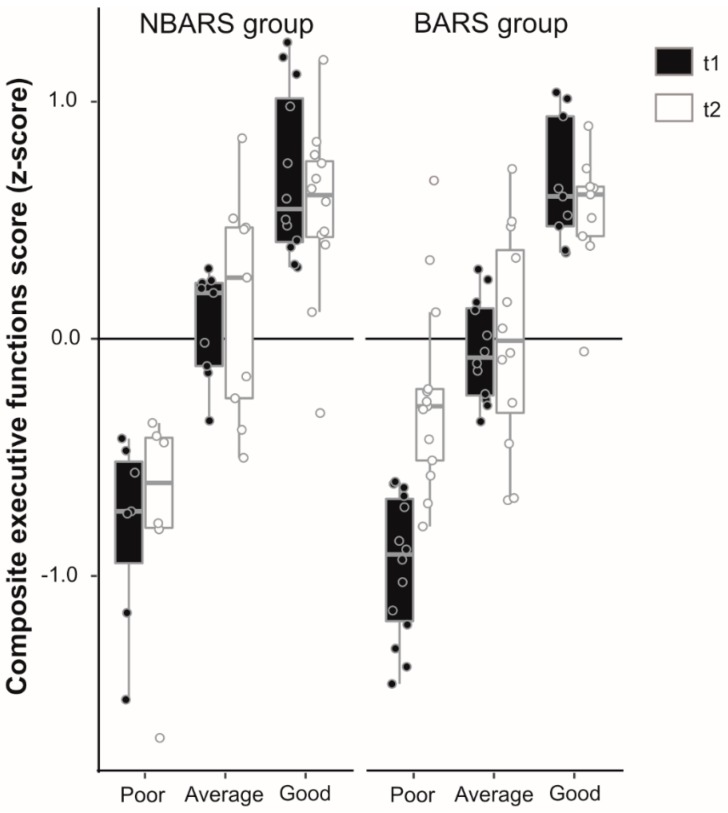
Boxplots (with median values and 95% confidence intervals) showing composite executive functions scores (*z*-scores) at t1 and t2 in the NBARS group (*n* = 29) compared with the BARS group (*n* = 40) both split in subjects who showed poor, average, or good performance at t1 (post-hoc analysis). Please note the significant increase in poor performers in the BARS group.

To explore whether our hypothesis stating that BARS compared to NBARS patients improve in executive functions might still be true for poor performers at t1, we split our sample into patients who scored high, average, and low in executive functions tests at t1 and estimated marginal effects in each group. This post-hoc analysis confirmed that subjects with poor executive control improved after BARS compared to patients in the NBARS group [I–J = 0.42, 95% CI = (0.1, 0.7), η_p_^2^ = 0.11, *p* = 0.01; see Figure 2], which was not the case for average [I–J = 0.17, 95% CI = (−0.1, 0.4), η_p_^2^ = 0.04, *p* = 0.14], and good performers [I–J = 0.02, 95% CI = (−0.24, 0.27), η_p_^2^ = 0.00, *p* = 0.90]. As hypothesized, a number of scores indicating mood/affect, and health-related quality of life showed an effect of surgery. In particular, state anxiety and depressive symptoms were lowered in the BARS compared to the NBARS group, while physiologic health-related quality of life improved, and patients reported more frequent physical activities (see Table 2). Within group comparisons confirmed this result and showed that surgery patients improved in all these variables, while NBARS patients even reported less physical activity at the follow up investigation [mean change = −0.65, t(19) = 2.29, *p* = 0.03, 95% CI = (0.06, 1.24)].

To investigate the question of whether improvement in executive functions was related to changes in insulin-glucose metabolism or inflammatory markers, exploratory correlation analyses in patients of the BARS group were computed. However, there were no substantial correlations between changes in blood parameters (i.e., fasting glucose, HbA1c, fasting insulin, HOMA-Index, TNF-α, IL-6, hsCRP) and executive functions scores.

### 3.3. Changes in GM Volume

**Figure 3:**
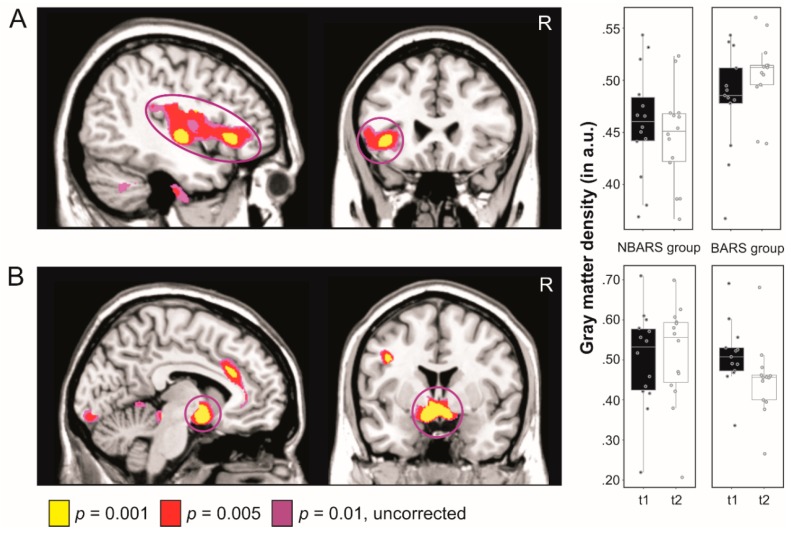
Changes in GM volume over time in the BARS group (*n* = 13) compared with the NBARS group (*n* = 14). **Left panel:** (**A**) Yellow-red brain regions show an increase in GM volume over time in the BARS compared to the NBARS group (contrast: BARS group > NBARS group^t2>t1^). (**B**) Yellow-red brain regions show a decrease in gray matter volume over time in the BARS compared to the NBARS group (contrast: BARS group > NBARS group^t1>t2^). **Right panel:** Boxplots (with median values and 95% confidence intervals) showing GM volume in arbitrary units plotted for both groups in the left superior temporal gyrus extending to insula and inferior frontal gyrus (**A**) and the bilateral ventral striatum (**B**).

Analyzing changes in GM volume, we found a group x time interaction, indicating an increase of volume over time in the BARS compared to the BARS group (BARS group > NBARS group^t2>t1^) in the left superior temporal gyrus, extending to insula and inferior frontal gyrus (for coordinates, see Table 3(a) and Figure 3).

In the complementary contrast (BARS group > NBARS group^t1>t2^), there were no substantial GM changes after cluster-wise FWE-correction. Applying a voxel-wise small volume FWE-correction (SVC) using an a priori defined ROI of the bilateral nucleus accumbens; however, we found decreased GM volume over time in BARS patients as compared to the NBARS group (group × time interaction; for intragroup comparisons: t2 > t1 and t1 > t2 in the BARS and NBARS groups, see Table 3(b) and (c).

### 3.4. Differences between Surgical Procedures

The ANCOVAs testing for differences between patients who underwent either RYGB (combined restrictive-malabsorptive, RYGB group) or VSG/GB (purely restrictive, VSG/GB group) surgery, only revealed few substantial group × time interactions, indicating a greater weight loss (weight and BMI) in patients of the RYGB compared to the VSG/GB group (see Table 4). Patients of the RYGB group also showed a greater decrease in fasting insulin, HOMA-index, and total cholesterol than VSG/GB patients.

Surgery groups, however, did not differ substantially with regard to changes in cognitive functions, mood/affect, and health-related quality of life. In contrast to our hypotheses, exploratory within-group comparisons showed a number of changes in the VSG/GB group [an increase in the executive functions test score: Mean change = 0.30, 95% CI = (1.80, 2.87), t(17) = −2.40, *p* = 0.03, a decrease in negative affect: Mean change = −1.04, 95% CI = (1.80, 2.87), t(22) = 2.87, *p* = 0.01, as well as a decrease in state and trait anxiety: Mean change = −5.17, 95% CI = (1.44, 8.90), t(22) = 2.88, *p* = 0.01 and mean change = −7.27, 95% CI = (2.29, 12.24), t(14) = 3.13, *p* = 0.01], which were not seen in the RYGB group. Finally, the subgroup analysis did not reveal substantial baseline value × group interactions in any of the cognitive domains, in contrast to what could be seen in the BARS vs. NBARS group comparisons.

## 4. Discussion

In this longitudinal observational study, we investigated the effects of BARS on cognitive functions, mood, health-related quality of life, as well as on serum and anthropometric parameters. To further study the neural correlates underlying the putative changes in brain health, we analyzed changes in GM volume in a subsample eligible for MRI scanning and compared patients who underwent either RYGB (combined restrictive-malabsorptive) or VSG/GB (purely restrictive) surgery.

The study yielded three main results: First, no important differences in cognitive functions between BARS and NBARS group emerged; however, exploratory analyses revealed improvement in executive functions, particularly in patients who performed poorly at baseline. In addition to previously well-documented changes in weight, body fat proportions, and serum parameters of glucose metabolism and inflammation, we found significant improvements in mood/affect and quality of life in patients who underwent BARS as compared to the waiting list controls. Second, BARS patients showed an increase of GM volume in fronto-temporal brain areas accompanied by a reduction of volume in the nucleus accumbens. Third, we found that patients who underwent RYGB surgery had greater weight loss as compared to patients with purely restrictive treatment, yet did not benefit more in terms of cognitive performance. In addition, no association was found between cognitive changes and changes in glucose metabolism or inflammation parameters.

### 4.1. Effects of BARS on Cognitive Functions

In the present study, we did not find any substantial improvement in cognitive functions (primary and secondary outcomes) in patients of the BARS as compared to the NBARS group. This result was surprising in light of previous studies demonstrating the beneficial effects of weight loss after BARS on cognitive performance [19,20]. The only indication that BARS might have a beneficial effect on executive control [15] was seen in the exploratory post-hoc within-group analysis, and a between-group comparison, including subjects with low performance at baseline only, which could also be a statistical “regression to the mean”. The present study could also not replicate the finding of weight loss-induced recognition memory improvement reported in previous studies of our group (cf. References [9,53]).

The lack of substantial changes in cognitive scores might be due to the fact that we used a test battery mainly constructed for clinical use (i.e., for identifying older patients with mild cognitive impairment). Patients in our study, however, were rather young (mean age = 45.5 years, range from 26 to 68) and demonstrated baseline performance in the normal to high-normal range, which might have obscured improvements. The hypothesis that more sensitive experimental tasks might have yielded differential changes between groups is supported by the post-hoc analysis showing improvement in executive functions in patients who performed poorly at baseline.

Despite the lack of significant changes in cognitive scores, we found surgery-related improvement in mood, affect, and health-related quality of life. In particular, state anxiety and depressive symptoms were decreased, while physiologic well-being and physical activity increased. The reduction in depressive symptoms and anxiety are known to accompany weight loss in overweight individuals [54], and might be related to the experience of success in losing weight.

Although we noted improvements after BARS in several physical parameters, indicating improvements in cardiovascular risk factors (e.g., decreases in total weight, systolic and diastolic blood pressure, and improvements in glucose metabolism and inflammation), we were not able to link these changes to the improvements in executive functions (cf. References [23,55]).

### 4.2. Effects of BARS on Brain Structure

With regard to brain structural changes, we found an increase in GM volume in the left superior temporal gyrus, extending to insula and inferior frontal gyrus over time in the BARS as compared to the NBARS group (group × time interaction). Increased GM volume in patients of the BARS group might reflect increased synaptic connectivity and dendritic arborization, which has also been observed following cognitive and behavioral training interventions [56,57]. Similar changes have been reported after intense weight loss following a low-caloric diet [9], and after BARS [58,59]. Tuulari et al. [58], for instance, found an increase of global white matter density six months after surgery, as well as an increase of GM volume in occipital and inferior temporal brain regions. Another study by Liu et al. [60] investigated cortical thickness before and one month after BARS and reported a number of increases and decreases in various brain regions, which are thought to be implicated in executive control and self-referential processing, including the frontal, temporal, and cingulate cortex.

Notably, substantial weight loss-induced changes in brain structure were found, although we were not able to provide evidence for changes in cognitive functions. This result underscores the usefulness of sensitive biomarkers when uncovering intervention-related improvements in brain health. Changes in GM volume and functional neural networks might become apparent some time before changes in downstream cognition occur [27,28]. To also detect changes in cognitive function scores, we, therefore, suggest conducting future studies, including later follow-up measurements.

In addition to expected changes in fronto-temporal brain regions, we found a decrease of GM volume in the bilateral ventral striatum (i.e., the nucleus accumbens) in the BARS compared to the NBARS group. Since brain structures of the mesolimbic reward system are increased in volume in obesity (e.g., Reference [61]), (for a review, see Reference [62]), a decrease after BARS could indicate changes that accompany a modified and more healthy eating behavior. Eating fewer calories overall, and smaller portions, reduces the chronically enhanced neural activity in these brain regions [63,64], which will translate into a decrease of volume over time.

### 4.3. Influence of Surgical Procedure on Cognitive Performance

BARS provides an excellent interventional approach for studying the effects of weight loss on brain health. As described in the Introduction, BARS comprises different surgical procedures using either purely restrictive (VSG/GB) or combined restrictive- malabsorptive approaches (RYGB). Since patients treated with RYGB demonstrate greater and more rapid weight loss than patients treated with other procedures [22], beneficial effects of BARS on cognitive functions should be greater in the RYGB compared to the VSG/GB group. In line with this assumption, we found differences in weight, BMI, and glucose metabolism. However, no differences between groups over time in cognitive test scores were noted. We speculate that the lack of evidence for greater cognitive benefits of RYGB surgery might be related to greater side effects of this approach: Due to the strong modification of the gastrointestinal tract, patients face several restrictions (e.g., life-long vitamin supplementation and the need to follow strict dietary guidelines), which may considerably impair their quality of life [65,66]. The hypothesis that small cognitive improvements might be masked by side effects still needs to be evaluated in further studies. The lack of influence of the amount of weight loss on cognitive performance, moreover, is in line with previous studies of our group demonstrating improved cognitive performance already after moderate weight loss [53].

### 4.4. Limitations

Some limitations should be considered when interpreting our findings. First, the sample size in our study was rather small; although comparable to sample sizes from previous studies [15,67,68,69]. While including 40 subjects in the BARS group, we were only able to analyze the data of 29 subjects in the waiting list control group, due to recruitment difficulties and many drop-outs, especially in the NBARS group (BARS: *n* = 5; NBARS: *n* = 7). Therefore, we cannot fully exclude the possibility that a lack of statistically significant changes between t1 and t2 in cognitive parameters is due to the small number of participants. Thus, further studies are needed to replicate our results in larger samples. In particular, the analysis comparing cognitive outcomes between surgical procedures must be considered as preliminary. Moreover, the size of the MRI sample in our study was too small for computing correlational analyses or subgroup analyses comparing the two surgical groups. Since many patients did not fit into the scanner, we recommend using a scanner with a larger bore than the one implemented in the Siemens Trio system in further studies.

Second, it must be noted that our study population was rather heterogeneous. As described in Section 2, on methods, we included men and women with an age range from 26 to 68 years. To control for the effects of some covariates, we entered age, BMI, and baseline test scores in the statistical analyses. However, we still cannot exclude that improvements in cognitive scores were not detected because they were overlaid by sample heterogeneities.

Third, the present study is an observational study in which no blinding (neither of patients nor investigators) was possible. Moreover, subjects in the waiting list control group might have been frustrated, since their application for cost coverage by the health insurance company was still not approved, and they struggled with bureaucratic obstacles to attain this approval. Therefore, we cannot rule out that group-specific improvement in executive functions after surgery might be in part, due to motivation deficits in the waiting list control group. However, given that improvements in executive functions were also evident in paired t-tests within the BARS group, and paralleled by changes in brain structure, we consider this explanation as unlikely.

### 4.5. Conclusion and Outlook

Obesity represents a major health problem, since it increases the risk for a number of adverse conditions, including type 2 diabetes, coronary heart disease, stroke, and dementia. Since BARS leads to rapid and sustainable weight loss, it might be an excellent approach to promote not only cardiovascular, but also brain health. In the present longitudinal observational study, we analyzed changes in a comprehensive neuropsychological test battery in patients six months after BARS compared to a well-matched waiting list control group during a similar period of time. We found expected beneficial changes in weight and serum parameters, as well as substantial group differences in brain structure. These changes, however, did not (yet) translate into improved cognition, although we found some indication for improved executive control after BARS, particularly in low baseline performers. Future studies should evaluate the benefits of effectively treating obesity on brain health with more sensitive measures and longer follow-up periods. To specifically investigate the effect/advantage of BARS compared to other means inducing weight loss on brain health, future studies should include an additional study group of patients that lose weight through a lifestyle modification program.

Moreover, our study investigated for the first time, in an exploratory approach, whether different surgical procedures (purely restrictive vs. combined restrictive-malabsorptive) vary in long-term cognitive outcomes. As expected, RYGB patients demonstrated greater weight loss than patients with restrictive treatment alone. However, they did not benefit more in terms of cognitive functions. Given that no differences in cognition over time between surgical procedures emerged, our study does not support combined restrictive-malabsorptive approaches, that offer larger weight loss and more improvement on glucose metabolism, but also carry greater risks and disadvantages, such as lifelong vitamin supplementation and the need to follow strict diet guidelines to avoid vomiting and diarrhea [10,70]. Since the sample size was rather small, in particular for computing the subgroup analysis, further studies with greater sample sizes are needed to replicate this result.

## Figures and Tables

**Table 1:** Baseline characteristics [mean (SD)] (a) for the whole sample (NBARS vs. BARS group), (b) for the MRI sample (NBARS vs. BARS group), and (c) for the comparison of the different surgical procedures (purely restrictive: VSG/GB group vs. combined restrictive-malabsorptive: RYGB group). Changes with *p* ≤ 0.05 between groups are indicated by underscoring the numbers.

**(a) Whole Sample**			
	**NBARS Group**	**BARS Group**	***p***
*n*	29	40	
Age (years)	45 (12)	46 (11)	0.61
Gender (% women)	55.2	70.0	0.21 ^a^
Weight (kg)	133.2 (22.6)	140.9 (29.3)	0.24
BMI (kg/m^2^)	44.6 (5.7)	49.2 (7.7)	0.01
Education (years)	16 (3)	15 (4)	0.66
Verbal intelligence (vocabulary test score)	29.8 (3.4)	29.7 (3.11)	0.66
BDI (Beck’s depression inventory score)	14.6 (7.3) ^c^	13.1 (9.3) ^b^	0.51
Fasting glucose (mg/dL)	117.8 (33.2) ^d^	127.4 (62.5)	0.46
HbA1c (%)	6.1 (1.0) ^d^	6.3 (1.1)	0.44
**(b) MRI Sample**			
	**NBARS Group**	**BARS Group**	***p***
*n*	14	13	
Age (years)	47 (12)	41 (11)	0.15
Gender (% women)	57.1	84.6	0.12 ^a^
Weight (kg)	123.7 (14.9)	123.8 (16.4)	0.98
BMI (kg/m^2^)	42.5 (4.7)	44.5 (5.6)	0.30
Education (years)	15.5 (2.3)	15.3 (3.0)	0.88
Verbal intelligence (vocabulary test score)	30.2 (3.3)	29.5 (2.5)	0.56
BDI (Beck’s depression inventory score)	16.2 (6.6) ^d^	17.3 (13.2)	0.78
Fasting glucose (mg/dL)	113.8 (26.6) ^d^	130.8 (93.6)	0.54
HbA1c (%)	6.0 (1.1) ^d^	6.2 (1.1)	0.70
**(c) Comparison of Surgical Procedures**			
	**VSG/GB Group**	**RYGB Group**	***p***
*n*	23	17	
Age (years)	47 (11)	45 (10)	0.71
Gender (% women)	57	88	0.03 ^a^
Weight (kg)	147.8 (29.3)	131.6 (27.5)	0.08
BMI (kg/m^2^)	50.2 (8.5)	47.7 (6.5)	0.31
Education (years)	15 (4)	16 (4)	0.29
Verbal intelligence (vocabulary test score)	30.2 (2.5)	29.1 (3.8)	0.25
BDI (Beck’s depression inventory score)	14.0 (9.1) ^d^	11.9 (9.7) ^d^	0.51
Fasting glucose (mg/dL)	133.8 (79.2)	118.7 (27.7)	0.46
HbA1c (%)	6.3 (1.1)	6.2 (1.0)	0.66

^a^ Calculated with χ^2^-test. ^b^ Data was not available for two subjects. ^c^ Data was not available for five subjects. ^d^ Data was not available for one subject.

**Table 2:** Changes within and between NBARS and BARS groups [mean (SD) at t1 and t2]. Changes with *p* ≤ 0.05 within and between groups are indicated by underscoring the numbers.

	Within Groups						Between Groups			
	NBARS Group (*n* = 29)			BARS Group (*n* = 40)			Results of the Multiple Linear Regression			
	t1	t2	*p*	t1	t2	*p*	ß	95% CI	η_p_^2^	*p*
**Anthropometric Parameters**										
Weight (kg)	133.2 (22.6)	133.5 (21.6)	0.81	141.0 (29.7)	111.0 (25.6)	<0.001 ^a^	−28.5	(−33.0, −23.9)	0.72	<0.001
BMI (kg/m^2^)	44.6 (5.7)	44.7 (5.3)	0.84	49.3 (7.8)	38.8 (7.4)	<0.001 ^a^	−9.9	(−11.3, −8.4)	0.74	<0.001
Body fat percentage (%)	44.6 (8.1)	43.9 (7.9)	0.37 ^a^	46.6 (9.5)	40.3 (7.8)	<0.001 ^a^	−6.1	(−8.3, −3.9)	0.34	<0.001
Systolic blood pressure (mm Hg)	133.3 (15.1)	130.9 (12.5)	0.27 ^b^	136.3 (15.0)	126.2 (11.8)	<0.001 ^c^	−7.1	(−12.2, −2.0)	0.12	0.01
Diastolic blood pressure (mm Hg)	90.4 (13.1)	88.2 (9.9)	0.23 ^b^	89.0 (10.0)	83.3 (10.0)	<0.001 ^d^	−4.3	(−8.6, 0.0)	0.07	0.05
**Serum levels**										
Fasting glucose (mg/dL)	119.2 (34.0)	114.9 (19.3)	0.40 ^c^	127.4 (62.5)	110.8 (41.0)	0.01	−10.3	(−17.4, −3.1)	0.12	0.01
HbA1c (%)	6.1 (1.0)	5.8 (0.6)	0.08 ^b^	6.3 (1.1)	5.9 (1.1)	<0.001	−6.1	(−14.7, 2.6)	0.03	0.17
Fasting insulin (µU/mL)	25.5 (17.4)	27.0 (13.2)	0.49 ^b^	29.2 (19.6)	12.4 (7.8)	<0.001 ^a^	−16.4	(−21.1, −11.8)	0.45	<0.001
HOMA-index	7.9 (6.5)	8.1 (4.5)	0.84 ^c^	9.1 (7.1)	3.3 (2.3)	<0.001 ^b^	−5.2	(−7.2, −3.3)	0.34	<0.001
Triacylglycerides (mg/dL)	150.0 (59.0)	155.7 (71.5)	0.53 ^b^	176.8 (82.1)	129.5 (42.5)	<0.001	−13.0	(−21.7, −4.3)	0.13	0.004
Total cholesterol (mg/dL)	191.9 (35.1)	185.6 (32.3)	0.22 ^b^	194.1 (36.3)	182.5 (36.3)	0.02	−6.4	(−20.5, 7.8)	0.01	0.37
LDL-to-HDL ratio	2.8 (1.2)	2.6 (1.0)	0.12 ^b^	2.6 (0.8)	2.4 (0.7)	0.01	−0.1	(−0.5, 0.2)	0.01	0.39
TNF-α (pg/mL)	7.5 (2.1)	8.0 (2.7)	0.22 ^b^	10.1 (4.0)	8.4 (3.7)	0.01 ^a^	−1.0	(−2.6, 0.6)	0.03	0.21
IL-6 (pg/mL	3.2 (1.5)	3.7 (2.0)	0.11 ^b^	3.6 (1.8)	2.8 (1.0)	<0.001 ^a^	−1.2	(−2.0, −0.4)	0.13	0.004
hsCRP (mg/L)	8.5 (6.9)	9.5 (8.1)	0.28 ^b^	12.9 (11.6)	7.0 (7.8)	<0.001	−19.7	(−27.8, −11.7)	0.28	<0.001
Leptin (ng/mL)	12.7 (7.6)	17.0 (11.5)	0.02 ^c^	22.7 (11.8)	12.7 (12.0)	<0.001 ^a^	−11.9	(−17.7, −6.2)	0.22	<0.001
**Test Scores**										
Executive functions (z-scores)	0.18 (0.6)	0.12 (0.7)	0.51 ^b^	−0.21 (0.7)	0.05 (0.5)	0.01 ^e^	0.2	(−0.1, 0.4)	0.04	0.13
Memory (z-scores)	0.09 (0.8)	0.15 (0.6)	0.67	−0.08 (0.9)	−0.08 (0.8)	0.95	−0.1	(−0.4, 0.2)	0.01	0.5
Sensomotoric speed (z-scores)	−0.18 (0.7)	−0.02 (0.8)	0.26 ^a^	0.02 (0.9)	0.12 (0.8)	0.3	0.1	(0.3, 0.4)	0	0.74
Attention (z-scores)	0.19 (1.2)	0.12 (1.1)	0.73	−0.15 (0.9)	−0.07 (0.9)	0.6	−0.05	(−0.5, 0.4)	0	0.84
PANAS +	29.14 (6.9)	29.55 (5.8)	0.63	32.26 (7.5)	32.54 (7.6)	0.77 ^a^	0.1	(−2.4, 2.6)	0	0.94
PANAS −	14.07 (4.9)	13.90 (4.2)	0.81	11.90 (1.6)	11.73 (0.7)	0.7	0.1	(−1.5, 1.6)	0	0.91
STAI state anxiety	36.93 (88.5)	38.83 (8.8)	0.22	36.53 (9.1)	33.00 (7.5)	0.02	−4.7	(−8.3, −1.1)	0.1	0.01
STAI trait anxiety	44.18 (11.9)	43.09 (13.2)	0.53 ^f^	43.33 (11.0)	38.13 (10.6)	0.02 ^g^	−3.4	(−8.5, 1.7)	0.04	0.18
BDI	14.57 (7.5)	13.87 (10.4)	0.63 ^e^	13.24 (9.9)	6.39 (8.1)	<0.001 ^f^	−6.5	(−10.4, −2.5)	0.18	0.002
SF-12 physiologic	39.44 (11.2)	40.23 (11.4)	0.67 ^g^	34.31 (10.8)	45.21 (10.3)	<0.001 ^g^	9.2	(4.0, 14.3)	0.23	0.001
SF-12 psychologic	45.65 (11.8)	45.81 (12.8)	0.96 ^g^	47.27 (12.4)	51.18 (11.6)	0.18 ^g^	1.3	(−5.4, 8.0)	0	0.7
FKA	2.70 (1.6)	2.05 (1.1)	0.03 ^h^	2.58 (1.5)	2.84 (1.2)	0.26 ^h^	0.9	(0.2, 1.5)	0.15	0.01

^a^ Data was not available for one subject. ^b^ Data was not available for two subjects. ^c^ Data was not available for three subjects. ^d^ Data was not available for four subjects. ^e^ Data was not available for six subjects. ^f^ Data was not available for seven subjects. ^g^ Data was not available for ten subjects. ^h^ Data was not available for nine subjects.

**Table 3:** Results of the whole-brain voxel-based GM volume analyses showing (a) group × time interactions and within-group comparisons over time (b) and (c); BARS group: *n* = 13; NBARS group: *n* = 14). Reported clusters were cluster-wise FWE-corrected at *p* < 0.05 at a cluster-defining uncorrected threshold of *p* < 0.005.

Anatomical Region	L/R	Number of Voxels in Cluster	*Z* score of Local Maximum	MNI Peak Voxel Coordinates		
				x	y	z
**(a) Group x Time interactions**						
*BARS group > NBARS group^t2>t1^* *(indicating an increase of volume in the surgery compared to the control group)*						
Superior temporal gyrus, insula, inferior frontal gyrus	L	3566	3.97	−44	−13	−3
*BARS group > NBARS group^t1>t2^* *(indicating a decrease of volume in the surgery compared to the control group)*						
Nucleus accumbens *	L	78	3.32	−8	6	−6
**(b) Within surgery group comparisons**						
*BARS group^t2>t1^ (indicating an increase)*						
Middle occipital gyrus, supramarginal gyrus	L	3175	3.74	−30	−70	25
*BARS group^t1>t2^ (indicating a decrease)*						
No ROI, nucleus putamen	L	8877	4.84	−9	4	−15
Supramarginal gyrus, cerebellum	R	2586	4.43	62	−42	30
No ROI, medial inferior occipital cortex	L	3004	3.85	−26	−102	−9
**(c) Within control group comparisons**						
*NBARS group^t2>t1^ (indicating an increase)*	No suprathreshold clusters					
*NBARS group^t1>t2^ (indicating a decrease)*	No suprathreshold clusters					

* Voxel-wise small volume corrected (SVC) using an a priori defined ROI of the bilateral nucleus accumbens obtained from the Automated Anatomical Labeling ROI library (AAL; [52]). L = left hemisphere; R = right hemisphere.

**Table 4:** Changes within and between VSG/GB and RYGB groups [mean (SD) at t1 and t2]. Changes with *p* ≤ 0.05 within and between groups are indicated by underscoring the numbers.

	Within Groups						Between Groups			
	VSG/GB Group (*n* = 23)			RYGB Group (*n* = 17)			Results of the Multiple Linear Regression			
	t1	t2	*p*	t1	t2	*p*	ß	95% CI	η_p_^2^	*p*
**Anthropometric Parameters**										
Weight (kg)	148.3 (29.8)	120.0 (24.6)	<0.001 ^a^	131.6 (27.5)	99.44 (22.4)	<0.001	−6.7	(−12.8, −0.6)	0.13	0.03
BMI (kg/m^2^)	50.5 (8.6)	41.0 (7.9)	<0.001 ^a^	47.7 (6.5)	36.0 (5.6)	<0.001	−2.3	(−4.2, −0.4)	0.15	0.02
Body fat percentage (%)	44.3 (11.3)	39.4 (9.2)	0.001 ^a^	49.5 (5.2)	41.4 (5.6)	<0.001	−0.8	(−3.7, 2.1)	0.01	0.59
Systolic blood pressure (mm Hg)	135.3 (13.8)	128.1 (10.5)	0.02 ^a^	137.7 (17.1)	123.6 (13.4)	<0.001 ^b^	−5.4	(−12.0, 1.2)	0.08	0.11
Diastolic blood pressure (mm Hg)	87.6 (9.8)	84.2 (9.7)	0.06 ^a^	90.0 (10.7)	81.5 (10.3)	0.002 ^b^	−4.2	(−9.9, 1.5)	0.07	0.14
**Serum Levels**										
Fasting glucose (mg/dL)	133.8 (79.2)	113.8 (49.3)	0.02	118.7 (27.7)	106.7 (27.0)	0.18	1.1	(−3.7, 5.9)	0.01	0.65
HbA1c (%)	6.3 (1.1)	6.0 (1.2)	0.001	6.2 (1.0)	5.8 (0.8)	0.003	−0.1	(−0.4, 0.2)	0.01	0.56
Fasting insulin (µU/mL)	29.0 (20.1)	13.7 (8.5)	<0.001	29.6 (19.6)	10.27 (6.2)	<0.001 ^a^	−3.8	(−6.9, −0.7)	0.16	0.02
HOMA-index	8.8 (6.7)	3.7 (2.1)	<0.001 ^a^	9.4 (7.8)	2.8 (2.5)	<0.001 ^a^	−3.0	(−4.8, −1.2)	0.26	0.002
Triacylglycerides (mg/dL)	200.1 (91.6)	141.7 (45.2)	0.01	145.2 (55.3)	112.9 (32.9)	<0.001	−2.5	(−7.8, 2.7)	0.03	0.34
Total cholesterol (mg/dL)	197.9 (40.4)	193.7 (35.9)	0.49	189.0 (30.1)	167.4 (32.0)	0.01	−20.6	(−38.7, −2.5)	0.13	0.03
LDL-to-HDL ratio	2.7 (0.8)	2.5 (0.7)	0.16	2.5 (0.9)	2.2 (0.7)	0.04	−0.2	(−0.6, 0.1)	0.04	0.22
TNF-α (pg/mL)	10.3 (3.7)	8.3 (3.0)	0.03	9.7 (4.4)	8.6 (4.6)	0.07 ^a^	0.8	(−1.3, 2.9)	0.02	0.45
IL-6 (pg/mL	3.7 (2.1)	2.8 (1.1)	0.02	3.4 (1.3)	2.7 (0.9)	0.02 ^a^	−1.1	(−6.5, 4.4)	0.01	0.69
hsCRP (mg/L)	12.6 (10.8)	8.0 (8.6)	0.003	13.3 (13.0)	5.5 (6.4)	0.003	−2.9	(−8.7, 3.0)	0.03	0.32
Leptin (ng/mL)	23.7 (13.3)	15.2 (13.6)	0.002 ^a^	21.4 (9.7)	9.4 (8.7)	<0.001	−4.4	(−11.0, 2.1)	0.05	0.18
**Test Scores**										
Executive functions (z-scores)	−0.10 (0.7)	0.20 (0.6)	0.03 ^c^	−0.15 (0.8)	0.06 (0.5)	0.17 ^a^	−0.2	(−0.4, 0.1)	0.04	0.3
Memory (z-scores)	−0.23 (0.8)	−0.23 (0.8)	0.98	0.31 (0.9)	0.32 (0.5)	0.96	5.1	(−1.6, 11.7)	0.07	0.13
Sensomotoric speed (z-scores)	−0.07 (1.0)	0.03 (0.8)	0.92	−0.01 (0.7)	0.07 (0.7)	0.88	−0.0	(−0.4, 0.3)	0	0.84
Attention (z-scores)	−0.22 (1.0)	−0.20 (0.9)	0.92	0.19 (0.9)	0.37 (1.1)	0.47	0.4	(−0.2, 1.0)	0.05	0.21
PANAS +	33.04 (8.2)	33.96 (8.6)	0.5	31.13 (6.5)	30.50 (5.6)	0.63 ^a^	−2.2	(−5.7, 1.3)	0.05	0.22
PANAS −	12.09 (1.8)	11.04 (1.5)	0.01	11.65 (1.3)	12.65 (4.0)	0.27	5.4	(−1.0, 11.7)	0.08	0.1
STAI state anxiety	35.96 (9.3)	30.78 (7.2)	0.01	37.29 (9.1)	36.00 (6.9)	0.55	3.8	(−0.5, 8.1)	0.09	0.08
STAI trait anxiety	43.20 (12.5)	35.93 (10.4)	0.01	42.71 (9.6)	41.43 (10.2)	0.58 ^d^	5.8	(−0.5, 12.0)	0.13	0.07
BDI	14.50 (10.6)	6.88 (8.8)	0.001	11.94 (9.7)	6.25 (7.7)	0.01 ^a^	−0.2	(−4.6, 4.2)	0	0.92
SF-12 physiologic	31.72 (8.1)	45.61 (8.7)	<0.001	38.07 (13.1)	43.93 (12.2)	0.04 ^e^	−5.4	(−11.9, 1.0)	0.11	0.1
SF-12 psychologic	47.55 (13.5)	51.98 (11.4)	0.23	48.00 (11.3)	49.69 (12.5)	0.57 ^e^	−0.9	(−8.3, 6.5)	0	0.8
FKA	2.94 (1.6)	3.19 (1.4)	0.43	2.20 (1.4)	2.47 (1.0)	0.43 ^b^	−0.3	(−1.1, 0.5)	0.02	0.47

^a^ Data was not available for one subject. ^b^ Data was not available for two subjects. ^c^ Data was not available for five subjects. ^d^ Data was not available for three subjects. ^e^ Data was not available for four subjects.

## Data Availability

The datasets used and analyzed in the current study are available from the corresponding author on reasonable request.

## References

[1] Finucane M.M., Stevens G.A., Cowan M.J., Danaei G., Lin J.K., Paciorek C.J., Singh G.M., Gutierrez H.R., Lu Y., Bahalim A.N. (2011). National, regional, and global trends in body-mass index since 1980: Systematic analysis of health examination surveys and epidemiological studies with 960 country-years and 9·1 million participants. Lancet.

[2] Guh D.P., Zhang W., Bansback N., Amarsi Z., Birmingham C.L., Anis A.H. (2009). The incidence of co-morbidities related to obesity and overweight: A systematic review and meta-analysis. BMC Public Health.

[3] Cournot M., Marquie J.C., Ansiau D., Martinaud C., Fonds H., Ferrieres J., Ruidavets J.B. (2006). Relation between body mass index and cognitive function in healthy middle-aged men and women. Neurology.

[4] Elias M.F., Elias P.K., Sullivan L.M., Wolf P.A., D’Agostino R.B. (2005). Obesity, diabetes and cognitive deficit: The Framingham Heart Study. Neurobiol. Aging.

[5] Fitzpatrick A.L., Kuller L.H., Lopez O.L., Diehr P., O’Meara E.S., Longstreth W.T., Luchsinger J.A. (2009). Midlife and late-life obesity and the risk of dementia: Cardiovascular health study. Arch. Neurol..

[6] Martin B., Mattson M.P., Maudsley S. (2006). Caloric restriction and intermittent fasting: Two potential diets for successful brain aging. Ageing Res. Rev..

[7] Mattson M.P. (2010). The impact of dietary energy intake on cognitive aging. Front. Aging Neurosci..

[8] Siervo M., Arnold R., Wells J.C., Tagliabue A., Colantuoni A., Albanese E., Brayne C., Stephan B.C. (2011). Intentional weight loss in overweight and obese individuals and cognitive function: A systematic review and meta-analysis. Obes. Rev..

[9] Prehn K., Jumpertz von Schwartzenberg R., Mai K., Zeitz U., Witte A.V., Hampel D., Szela A.M., Fabian S., Grittner U., Spranger J. (2017). Caloric Restriction in Older Adults-Differential Effects of Weight Loss and Reduced Weight on Brain Structure and Function. Cereb. Cortex.

[10] Yu J., Zhou X., Li L., Li S., Tan J., Li Y., Sun X. (2015). The long-term effects of bariatric surgery for type 2 diabetes: Systematic review and meta-analysis of randomized and non-randomized evidence. Obes. Surg..

[11] Peirson L., Douketis J., Ciliska D., Fitzpatrick-Lewis D., Ali M.U., Raina P. (2014). Prevention of overweight and obesity in adult populations: A systematic review. CMAJ Open.

[12] Wittgrove A.C., Clark G.W., Tremblay L.J. (1994). Laparoscopic Gastric Bypass, Roux-en-Y: Preliminary Report of Five Cases. Obes. Surg..

[13] Alosco M.L., Galioto R., Spitznagel M.B., Strain G., Devlin M., Cohen R., Crosby R.D., Mitchell J.E., Gunstad J. (2014). Cognitive function after bariatric surgery: Evidence for improvement 3 years after surgery. Am. J. Surg..

[14] Gunstad J., Strain G., Devlin M.J., Wing R., Cohen R.A., Paul R.H., Crosby R.D., Mitchell J.E. (2011). Improved memory function 12 weeks after bariatric surgery. Surg. Obes. Relat. Dis..

[15] Marques E.L., Halpern A., Correa Mancini M., de Melo M.E., Horie N.C., Buchpiguel C.A., Martins Novaes Coutinho A., Ono C.R., Prando S., Santo M.A. (2014). Changes in neuropsychological tests and brain metabolism after bariatric surgery. J. Clin. Endocrinol. Metab..

[16] Miller L.A., Crosby R.D., Galioto R., Strain G., Devlin M.J., Wing R., Cohen R.A., Paul R.H., Mitchell J.E., Gunstad J. (2013). Bariatric surgery patients exhibit improved memory function 12 months postoperatively. Obes. Surg..

[17] Stroop J.R. (1935). Studies of interference in serial verbal reactions. J. Exp. Psychol..

[18] Helmstaedter C., Lendt M., Lux S. (2001). Verbaler Lern-und Merkfähigkeitstest (VLMT).

[19] Handley J.D., Williams D.M., Caplin S., Stephens J.W., Barry J. (2016). Changes in Cognitive Function Following Bariatric Surgery: A Systematic Review. Obes. Surg..

[20] Thiara G., Cigliobianco M., Muravsky A., Paoli R.A., Mansur R., Hawa R., McIntyre R.S., Sockalingam S. (2017). Evidence for Neurocognitive Improvement After Bariatric Surgery: A Systematic Review. Psychosomatics.

[21] Georgiadou E., Gruner-Labitzke K., Kohler H., de Zwaan M., Muller A. (2014). Cognitive function and nonfood-related impulsivity in post-bariatric surgery patients. Front. Psychol..

[22] Buchwald H., Avidor Y., Braunwald E., Jensen M.D., Pories W., Fahrbach K., Schoelles K. (2004). Bariatric surgery: A systematic review and meta-analysis. JAMA.

[23] Alosco M.L., Spitznagel M.B., Strain G., Devlin M., Cohen R., Crosby R.D., Mitchell J.E., Gunstad J. (2015). Improved serum leptin and ghrelin following bariatric surgery predict better postoperative cognitive function. J. Clin. Neurol..

[24] Ashrafian H., le Roux C.W. (2009). Metabolic surgery and gut hormones—A review of bariatric entero-humoral modulation. Physiol. Behav..

[25] Smith K.R., Moran T.H., Papantoni A., Speck C., Bakker A., Kamath V., Carnell S., Steele K.E. (2019). Short-term improvements in cognitive function following vertical sleeve gastrectomy and Roux-en Y gastric bypass: A direct comparison study. Surg. Endosc..

[26] Ainsworth B.E., Haskell W.L., Leon A.S., Jacobs D.R., Montoye H.J., Sallis J.F., Paffenbarger R.S. (1993). Compendium of physical activities: Classification of energy costs of human physical activities. Med. Sci. Sports Exerc..

[27] Kobe T., Witte A.V., Schnelle A., Lesemann A., Fabian S., Tesky V.A., Pantel J., Floel A. (2016). Combined omega-3 fatty acids, aerobic exercise and cognitive stimulation prevents decline in gray matter volume of the frontal, parietal and cingulate cortex in patients with mild cognitive impairment. Neuroimage.

[28] Prehn K., Lesemann A., Krey G., Witte A.V., Kobe T., Grittner U., Floel A. (2019). Using resting-state fMRI to assess the effect of aerobic exercise on functional connectivity of the DLPFC in older overweight adults. Brain Cogn..

[29] Nho K., Risacher S.L., Crane P.K., DeCarli C., Glymour M.M., Habeck C., Kim S., Lee G.J., Mormino E., Mukherjee S. (2012). Voxel and surface-based topography of memory and executive deficits in mild cognitive impairment and Alzheimer’s disease. Brain Imaging Behav..

[30] Folstein M.F., Folstein S.E., McHugh P.R. (1975). “Mini-mental state” A practical method for grading the cognitive state of patients for the clinician. J. Psychiatr. Res..

[31] Creavin S.T., Wisniewski S., Noel-Storr A.H., Trevelyan C.M., Hampton T., Rayment D., Thom V.M., Nash K.J., Elhamoui H., Milligan R. (2016). Mini-Mental State Examination (MMSE) for the detection of dementia in clinically unevaluated people aged 65 and over in community and primary care populations. Cochrane Database Syst. Rev..

[32] Lezak M.D. (2004). Neuropsychological Assessment.

[33] Reitan R.M., Wolfson D. (1993). The Halstead–Reitan Neuropsychological Test Battery: Theory and Clinical Interpretation.

[34] van de Rest O., Geleijnse J.M., Kok F.J., van Staveren W.A., Dullemeijer C., Olderikkert M.G., Beekman A.T., de Groot C.P. (2008). Effect of fish oil on cognitive performance in older subjects: A randomized, controlled trial. Neurology.

[35] Watson D., Clark L.A., Tellegen A. (1988). Development and validation of brief measures of positive and negative affect: The PANAS scales. J. Pers. Soc. Psychol..

[36] Spielberger C.D., Gorsuch R.L., Lushene R.E. (1970). Manual for the State-Trait Anxiety Inventory.

[37] Ware J., Kosinski M., Keller S.D. (1996). A 12-Item Short-Form Health Survey: Construction of scales and preliminary tests of reliability and validity. Med. Care.

[38] Mueller K., Moller H.E., Horstmann A., Busse F., Lepsien J., Bluher M., Stumvoll M., Villringer A., Pleger B. (2015). Physical exercise in overweight to obese individuals induces metabolic- and neurotrophic-related structural brain plasticity. Front. Hum. Neurosci..

[39] Frey I., Berg A., Grathwohl D., Keul J. (1999). [Freiburg Questionnaire of physical activity--development, evaluation and application]. Soz. Praventivmed..

[40] Kuhner C., Burger C., Keller F., Hautzinger M. (2007). Reliability and validity of the Revised Beck Depression Inventory (BDI-II). Results from German samples. Nervenarzt.

[41] Lehrl S. (2005). Mehrfachwahl-Wortschatz-Intelligenztest MWT-B.

[42] Tohka J., Zijdenbos A., Evans A. (2004). Fast and robust parameter estimation for statistical partial volume models in brain MRI. Neuroimage.

[43] Rajapakse J.C., Giedd J.N., Rapoport J.L. (1997). Statistical approach to segmentation of single-channel cerebral MR images. IEEE Trans. Med. Imaging.

[44] Cuadra M.B., Cammoun L., Butz T., Cuisenaire O., Thiran J.-P. (2005). Comparison and validation of tissue modelization and statistical classification methods in T1-weighted MR brain images. IEEE Trans. Med. Imaging.

[45] Ashburner J. (2007). A fast diffeomorphic image registration algorithm. Neuroimage.

[46] Freund W., Faust S., Gaser C., Gron G., Birklein F., Wunderlich A.P., Muller M., Billich C., Schutz U.H. (2014). Regionally accentuated reversible brain grey matter reduction in ultra marathon runners detected by voxel-based morphometry. BMC Sports. Sci. Med. Rehabil..

[47] Silver M., Montana G., Nichols T.E. (2011). False positives in neuroimaging genetics using voxel-based morphometry data. Neuroimage.

[48] Shen S., Sterr A. (2013). Is DARTEL-based voxel-based morphometry affected by width of smoothing kernel and group size? A study using simulated atrophy. J. Magn. Reson. Imaging.

[49] Hodgson K., Poldrack R.A., Curran J.E., Knowles E.E., Mathias S., Goring H.H.H., Yao N., Olvera R.L., Fox P.T., Almasy L. (2017). Shared Genetic Factors Influence Head Motion During MRI and Body Mass Index. Cereb. Cortex.

[50] Makowski C., Lepage M., Evans A.C. (2019). Head motion: The dirty little secret of neuroimaging in psychiatry. J. Psychiatry Neurosci..

[51] Zeng L.L., Wang D., Fox M.D., Sabuncu M., Hu D., Ge M., Buckner R.L., Liu H. (2014). Neurobiological basis of head motion in brain imaging. Proc. Natl. Acad. Sci. USA.

[52] Tzourio-Mazoyer N., Landeau B., Papathanassiou D., Crivello F., Etard O., Delcroix N., Mazoyer B., Joliot M. (2002). Automated anatomical labeling of activations in SPM using a macroscopic anatomical parcellation of the MNI MRI single-subject brain. Neuroimage.

[53] Witte A.V., Fobker M., Gellner R., Knecht S., Floel A. (2009). Caloric restriction improves memory in elderly humans. Proc. Natl. Acad. Sci. USA.

[54] Herpertz S., Muller A., Burgmer R., Crosby R.D., de Zwaan M., Legenbauer T. (2015). Health-related quality of life and psychological functioning 9 years after restrictive surgical treatment for obesity. Surg. Obes. Relat. Dis..

[55] Hawkins M.A., Alosco M.L., Spitznagel M.B., Strain G., Devlin M., Cohen R., Crosby R.D., Mitchell J.E., Gunstad J. (2015). The Association Between Reduced Inflammation and Cognitive Gains After Bariatric Surgery. Psychosom. Med..

[56] Trachtenberg J.T., Chen B.E., Knott G.W., Feng G., Sanes J.R., Welker E., Svoboda K. (2002). Long-term in vivo imaging of experience-dependent synaptic plasticity in adult cortex. Nature.

[57] Zatorre R.J., Fields R.D., Johansen-Berg H. (2012). Plasticity in gray and white: Neuroimaging changes in brain structure during learning. Nat. Neurosci..

[58] Tuulari J.J., Karlsson H.K., Antikainen O., Hirvonen J., Pham T., Salminen P., Helmio M., Parkkola R., Nuutila P., Nummenmaa L. (2016). Bariatric Surgery Induces White and Grey Matter Density Recovery in the Morbidly Obese: A Voxel-Based Morphometric Study. Hum. Brain Mapp..

[59] Rullmann M., Preusser S., Poppitz S., Heba S., Hoyer J., Schutz T., Dietrich A., Muller K., Pleger B. (2018). Gastric-bypass surgery induced widespread neural plasticity of the obese human brain. Neuroimage.

[60] Liu L., Ji G., Li G., Hu Y., Jin Q., Hu C., Zhao J., Meng Q., von Deneen K.M., Chen A. (2019). Structural changes in brain regions involved in executive-control and self-referential processing after sleeve gastrectomy in obese patients. Brain Imaging Behav..

[61] Horstmann A., Busse F.P., Mathar D., Muller K., Lepsien J., Schlogl H., Kabisch S., Kratzsch J., Neumann J., Stumvoll M. (2011). Obesity-Related Differences between Women and Men in Brain Structure and Goal-Directed Behavior. Front. Hum. Neurosci..

[62] Kenny P.J. (2011). Reward mechanisms in obesity: New insights and future directions. Neuron.

[63] Coveleskie K., Gupta A., Kilpatrick L.A., Mayer E.D., Ashe-McNalley C., Stains J., Labus J.S., Mayer E.A. (2015). Altered functional connectivity within the central reward network in overweight and obese women. Nutr. Diabetes.

[64] Rothemund Y., Preuschhof C., Bohner G., Bauknecht H.C., Klingebiel R., Flor H., Klapp B.F. (2007). Differential activation of the dorsal striatum by high-calorie visual food stimuli in obese individuals. Neuroimage.

[65] Backman O., Stockeld D., Rasmussen F., Naslund E., Marsk R. (2016). Alcohol and substance abuse, depression and suicide attempts after Roux-en-Y gastric bypass surgery. Br. J. Surg..

[66] Musselman D., Shenvi N., Manatunga A., Miller A.H., Lin E., Gletsu-Miller N. (2019). The effects of roux en y gastric bypass surgery on neurobehavioral symptom domains associated with severe obesity. Physiol. Behav..

[67] Ochner C.N., Kwok Y., Conceicao E., Pantazatos S.P., Puma L.M., Carnell S., Teixeira J., Hirsch J., Geliebter A. (2011). Selective reduction in neural responses to high calorie foods following gastric bypass surgery. Ann. Surg..

[68] van de Sande-Lee S., Pereira F.R., Cintra D.E., Fernandes P.T., Cardoso A.R., Garlipp C.R., Chaim E.A., Pareja J.C., Geloneze B., Li L.M. (2011). Partial reversibility of hypothalamic dysfunction and changes in brain activity after body mass reduction in obese subjects. Diabetes.

[69] Zhang Y., Ji G., Xu M., Cai W., Zhu Q., Qian L., Zhang Y.E., Yuan K., Liu J., Li Q. (2016). Recovery of brain structural abnormalities in morbidly obese patients after bariatric surgery. Int. J. Obes..

[70] Sherf Dagan S., Goldenshluger A., Globus I., Schweiger C., Kessler Y., Kowen Sandbank G., Ben-Porat T., Sinai T. (2017). Nutritional Recommendations for Adult Bariatric Surgery Patients: Clinical Practice. Adv. Nutr..

